# Production of Milk Phospholipid-Enriched Dairy Ingredients

**DOI:** 10.3390/foods9030263

**Published:** 2020-03-02

**Authors:** Zhiguang Huang, Haotian Zheng, Charles S. Brennan, Maneesha S. Mohan, Letitia Stipkovits, Lingyi Li, Don Kulasiri

**Affiliations:** 1 Department of Wine, Food and Molecular Biosciences, Faculty of Agriculture and Life Sciences, Lincoln University, Lincoln 7647, Christchurch, New Zealand; 2 Riddet Research Institute, Palmerston North 4442, New Zealand; 3 Department of Food, Bioprocessing and Nutrition Sciences, Southeast Dairy Foods Research Center, North Carolina State University, Raleigh, NC 27695, USA; 4 Dairy Innovation Institute, California Polytechnic State University, San Luis Obispo, CA 93407, USA; 5 Tianjin Key Laboratory of Food and Biotechnology, School of Biotechnology and Food Science, Tianjin University of Commerce, Tianjin 300134, China

**Keywords:** milk phospholipids, buttermilk, life-cycle assessment, carbon footprint, supercritical fluid extraction, membrane separation

## Abstract

Milk phospholipids (MPLs) have been used as ingredients for food fortification, such as bakery products, yogurt, and infant formula, because of their technical and nutritional functionalities. Starting from either buttermilk or beta serum as the original source, this review assessed four typical extraction processes and estimated that the life-cycle carbon footprints (CFs) of MPLs were 87.40, 170.59, 159.07, and 101.05 kg CO_2_/kg MPLs for membrane separation process, supercritical fluid extraction (SFE) by CO_2_ and dimethyl ether (DME), SFE by DME, and organic solvent extraction, respectively. Regardless of the MPL content of the final products, membrane separation remains the most efficient way to concentrate MPLs, yielding an 11.1–20.0% dry matter purity. Both SFE and solvent extraction processes are effective at purifying MPLs to relatively higher purity (76.8–88.0% *w*/*w*).

## 1. Introduction

Milk phospholipids (MPLs) consist of a subclass of polar lipids, namely glycerophospholipids and sphingolipids [1]. Glycerophospholipids comprise a glycerol moiety with two fatty acids esterified at positions *sn*-1 and *sn*-2 and a hydroxyl group at *sn*-3 position, linked to a phosphate group and a polar moiety [1]. The molecular structure of the latter determines the types of glycerophospholipids, namely phosphatidylcholine (PC), phosphatidylserine (PS), phosphatidylethanolamine (PE), phosphatidylinositol (PI), phosphatidyl-glycerol (PG), and phosphatidic acid (PA) [2]. Sphingolipids consist of a sphingosine backbone (2-amino-4-octadecene-1,3-diol) connected to a fatty acid via an amide bond and a polar head. Sphingomyelin (SM), a prominent subclass of sphingolipids, has a phosphocholine residue [1]. In raw bovine milk, the diameters of milk fat globules (MFGs) are around 0.2–15 µm; these MFGs are enveloped by an approximately 15-nm thick tri-layer MFG membrane (MFGM) [3,4]. The composition of MFGM is 30–75% polar lipids, and 25–70% protein, respectively [5]. MPLs lie within the MFGM constructing its backbone. MPLs represent 0.4–1% of the total milk lipids [6], which change with season, lactose stage, and feed [7].

MPLs have exhibited nutraceutical properties due the unique composition of this group of phospholipids. MPLs contain high proportions of SM [8] and PS [9] (24% and 12%, respectively), subclasses which are virtually absent in other sources, such as soy (0% and 0.5%, respectively) and egg yolk lecithin (1.5% and 0%, respectively) [10]. PS is associated with cognitive function and releasing stress, and is replaced by inactive cholesterol as the brain ages [11,12]. SM has been found to be effective in inhibiting colon tumors [13]. Also, MPLs have been implicated in mitigating the risks of Alzheimer’s disease and repairing cognitive ability [14], restoring immunological defenses, reducing the incidence of cardiovascular diseases [15,16], and reducing cholesterol absorption and total liver lipids [17]. In addition, MPLs may narrow the gap between formula-fed and breastfed infants concerning neurodevelopment, infectious diseases, and cholesterol metabolism [18,19]. Phospholipid-coated fats, e.g., human breast MFGs, will be properly digested and absorbed, not only due to the size of the MFGs, but also due to the ratio of MFGM proteins to phospholipids [20]. Bovine MPL-enriched ingredients may be used to produce breast milk analogs. For instance, one formula recipe consists of subclasses according to a weight-relative ratio of SM > PC > PE > PS > PI, with 21.1–29.7% SM and 10.2–13.3% PS (both based on total MPLs, similar to those of human breast milk (37.5% and 9%, respectively) [21]. Another infant formula comprises 150 mg/L MPLs [22], mimicking that of breast milk (15–20 mg/dL milk [21] and 0.3–1.0% of the total lipids [23]).

Aside from nutritional value and health benefits, MPLs may provide technical functionalities in food systems, for example, MPLs have been used in the preparation of liposomes [24] and constructing vesicles of bioactive compounds [25]; they are also food emulsifiers and surfactants, foaming agents, texture improvers for bakery goods, and may improve moisture retention for yogurt [26,27].

Many research works and reviews are available on fractionation from buttermilk (BM) and beta serum (BS) [26], isolating MFGs by washing and centrifugation [5], and the membrane separation of polar lipids [8]. However, there is no standard large-scale manufacturing process adopted by the dairy industry. This is due to many reasons. First, the native MFGM is fragile. Shear and turbulent fluid flow can cause damage to the MFGM [28]. These treatments are commonly involved in handling raw milk on farms, in transportation, in silos at manufacturing plants, and during cream separation. Damage to the MFGM may cause associated materials, including MPLs, to deplete from the native MFGs to the aqueous phase of milk. Therefore, more than half of MPLs in raw milk remains in skim milk [29,30]. Second, uncertainties and variables are involved in the MPL fractionation processes. For example, cream washing for removing non-MFGM associated proteins may be performed before butter churning for increased yield or the concentration of MPLs in the resulting BM, or in the retentate of BM after tangential filtration. However, the cream washing procedure may cause a significant change to the MPL composition in BM from unwashed cream [31,32]. Although the mechanism is not clear, it may relate to the physicality of different washing processes. Zheng’s group revealed that different washing procedures induce various degrees of damage to MFGM. Therefore, washing may alter the composition of MPL in the fat phase of the washed cream [4,33]. This review aimed to assess different dairy streams rich in MPLs, to evaluate their extraction processes, compare their process intensity and efficiency, and to estimate their life-cycle carbon footprints (CFs) using ISO 14067 and greenhouse gas (GHG) protocols.

## 2. Milk Phospholipid Extraction from Dairy Products

### 2.1. Dairy By-Products Rich in Phospholipids

Commercial MPL products are usually derived from dairy products, such as BM [34], BS [8], acid cheese whey BM [35,36], whey protein phospholipids concentrate (WPPC) [37], or whey BM [38]. The dairy streams in Table 1 comprise 2.29%–26.02% MPLs on a dry matter (DM) basis, varying with sources and processes.

**Figure 1:**
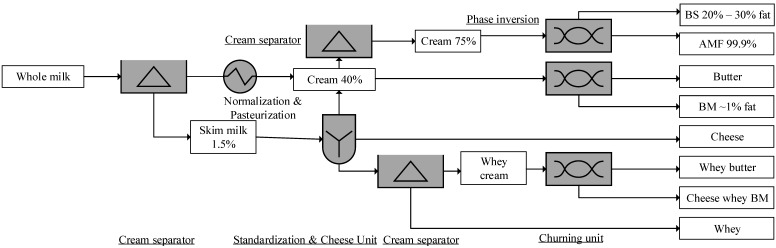
Buttermilk (BM) and beta serum (BS) production process [26]. AMF, anhydrous milk fat. % indicates the fat content.

BM is the product that remains after the removal of butter by churning cream, which may have been concentrated and/or dried as butter milk powder [39], as illustrated in Figure 1. Acid BM, a by-product of lactic butter, is made by churning cultured cream. Furthermore, whey BM is produced via the churning of whey cream during cheese making [40]. WPPC is a by-product produced during the microfiltration (MF) of whey for manufacturing whey protein isolate (WPI). The permeate phase (milk-fat-discriminated phase) from this process goes forward for WPI manufacturing and the fat-remaining phase (retentate phase) containing residual whey proteins is further concentrated for producing WPPC. A typical WPPC is comprises more than 12% fat and 50% protein (DM), and less than 8% ash and 6% moisture [37].

BM, the serum phase resulting from the churning of cream, comprises milk proteins and residual fat [34]. In terms of protein, lactose, ash, and DM contents, BS and BM are very similar to those of cream products (Table 1) [41]. For instance, BM (FDC ID 454974) protein content is 3.33%, which is the same as that of cream (FDC ID 495516). Though the fat content of BM is only one-tenth of cream, MPLs of BM are 4–27-fold that of raw milk, as shown in Table 1. The empirical equation MPL = 0.0137 × FC provides an estimation of the MPL content (g/L) of a dairy product, where FC is the fat content of cream [42]. For instance, the estimated BM MPL content of anhydrous milk fat (AMF) from 80% cream, and of butter from 40% cream, is 1.1 and 0.55 g/kg, respectively. Whey BM, a by-product of whey butter, comprises sixfold the MPL content of raw milk, as seen in Table 1 [38].

BM and BS, the most abundant source of MPLs [43], have been underexplored or even treated as a waste stream [44]. For instance, a New Zealand-based dairy manufacturer used two-thirds of their BM for standardization, only one-tenth for BM powder (BMP), and their annual output of MPL concentrate is 320 metric tons [44]. The annual BM output in Canada was 14.1 metric kilotons (18% of butter and 0.5% of bulk liquid [45]), compared to 20 kilotons in Belgium, 16 kilotons in Denmark, and 124.5 kilotons in Germany [46]. In 2013, approximately 5.2 million tons of BM was produced worldwide, similar to that of butter [34]. Worldwide, the annual BMP production was estimated at 410 kilotons (≈9.5% of butter), which has downstream applications for producing ice cream, ingredients-baked foods, low-fat Cheddar cheese, reduced-fat cheese, pizza cheese [40], or in the replacement of skim milk powder for low-fat yogurt [47].

### 2.2. Commercialized Milk Phospholipid Products and Concentrate

Phosphoric 500/600/700 and Gangolac 600 (products manufactured by Fonterra) comprise 34%, 75%, 62%, and 15% MPLs, respectively, representing one source of highly-purified MPLs [52,53]. Arla Foods Amba have developed phospholipid-rich, concentrated dairy milk commodities for infant milk formulas and skin care. It has been claimed that Lacprodan^®^ MFGM 10 supports physiological development of the infant gut and provides infants with similar health benefits to breast milk because of their similarities in fatty acid profile [54]. Arla dairy products PL 20/75 consist of 20% and 75% MPLs, respectively [55].

As illustrated in the patents in Table 2, both filtration and solvent extraction are validated processes for manufacturing MPLs. Acetone and supercritical CO_2_ are effective solvents for de-fatting. Tatua [56] and Synlait [57] have concentrated MPLs to 5–12.8% (*w*/*w*, DM basis). Lecico has used membrane separation to produce Lipamine M20 (20% purity) [58].

### 2.3. Laboratory Extraction of Milk Phospholipids

Intact MFGM makes up 2–6% of the total mass of MFG [26]. However, MFGM represents 60%–70% of the total milk phospholipids [69]. Raw bovine milk comprises 0.2–0.4 g MPLs/kg, and raw milk is generally a laboratory source of MPLs [5,70]. Intact MFGs can be isolated with low-speed centrifugation. The cream layer from raw milk skimming can be washed with phosphate buffered saline (PBS; pH 6.8, 0.1 M, 1:10, *v*/*v*) and centrifuged at 390 g for 10 min at 10 °C. The final cream layer after three washes is the large MFG fraction [71]. Different from isolating intact MFGM, Sanches-Juanes et al. [72] ruptured MFGM and recrystallized milk lipids, and starting from raw milk, they washed cream with a 0.15 M NaCl solution and precipitated casein using centrifugation at 5000× *g*.

Cream washing is a step used to remove casein and other non-MFGM materials from cream [44]. After centrifugation, casein will precipitate, with lipid stratification at the top layer [73]. Also, calcium, naturally present in casein micelles, can form a complex between MFGM and the casein micelles through its binding to the phospho-casein and phospholipids of MFGM, leaving impurities with MPLs [74]. In addition, washing causes a severe loss of phospholipids, almost 60% per wash [32]. Hence, washing facilities for separating MPLs are costly and energy-intensive [44], thereby they are mainly only used for laboratory purposes [5,73,75].

In addition to washing and centrifugation, the microfiltration of raw milk has been applied to produce MFGM material. It has been found that a 1.4-μm ceramic membrane was superior to 0.8 μm, yielding a high-purity MFGM material, which was composed of 7% phospholipids and 30% protein [76].

For analysis purposes, MPL samples are usually prepared using solvent extraction. The Folch [77] and Bligh [78] methods use chloroform and methanol to dissolve lipids. Other lipophilic extraction formulas include the Mojonnier solvents [79], dichloromethane [80], and the ammoniacal ethanolic solution of lipids with dimethyl ether and light petroleum in the Röse–Gottlieb extraction [81,82]. The total lipid content in samples can be determined with a gravimetric assay, Gerber-van Gulik butyrometer, infrared spectroscopy according to an International Dairy Federation (IDF) method [81], or gas chromatography equipped with a flame ionization detector [83].

To determine the MPLs and their subclasses, solid-phase extraction can fractionate polar lipids from non-polar lipids. Silica-gel-bonded cartridges or silica gel plates can be used for such a purpose [84]. The obtained MPLs can be solvent dried using a vacuum and stored at −20 °C before using [85]. In addition, chloroform and methanol are also valid elution solvents [86]. Total MPLs can be measured using the IDF molybdate assay [87], Fourier transform infrared spectroscopy [88], or a fluorescence assay on cleaved choline group [89]. Both nuclear magnetic resonance of ^31^P and chromatography can quantify MPLs and their subclasses [90,91]. High-performance liquid chromatography coupling with detectors as a charged aerosol detector, evaporative light-scattering detector, and mass spectroscopy is more acceptable than thin layer chromatography [92].

## 3. Processes for Industrial Manufacturing of Milk Phospholipids

### 3.1. Solvent Extraction

**Figure 2:**
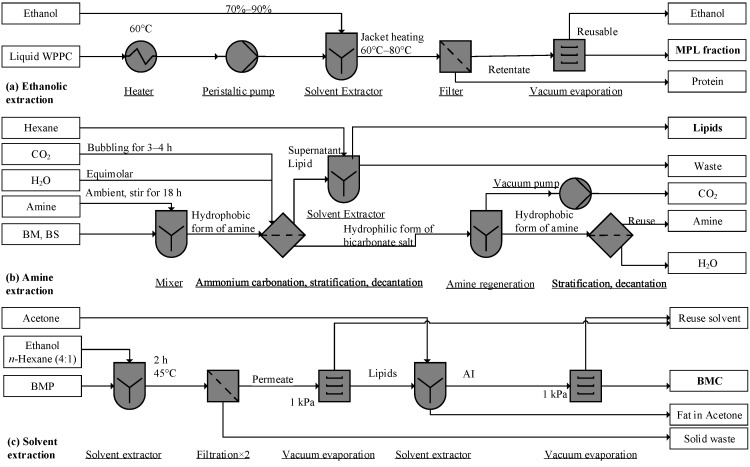
Process flow diagram of solvent extraction unit: (**a**) adapted from Price et al. [48], (**b**) Ota et al. [93], and (**c**) Shulman et al. [21]. BMC, buttermilk concentrate; MPL, milk phospholipid; AI, acetone insoluble; WPPC, whey protein phospholipid concentrate (liquid, reconstituted from powder).

Many kinds of polar solvents have been used to extract MPLs, such as ethanol and alkanes [21,66]. To separate casein from MPLs, proteins can also be thermally denatured or in an acid solution (pH 4.6) [48,81], the aggregated particles are subsequently filtrated. Regarding fractionation of MPL from WPPC, ethanol (70% *v*/*v*) at 60–80 °C denatures proteins; after filtration the MPL concentration is ≈45.8% in the filtrate in Figure 2a [48]. This notable method uses no toxic solvent. However, the incompleteness of the phospholipid recovery may be a concern [48].

Compared to 58.1% recovery by ethanol, the tertiary amine CyNMe2 (*N*,*N*-dimethylcycloexylamine) yielded a 99.96% recovery rate of MPLs. At various solvent–sample weight ratios, the lipid extraction was conducted at ambient temperature. The dissolved MPLs in the amine were released by bubbling CO_2_ at atmospheric pressure, which converts CyNMe2 into the carbonate salt in Figure 2b. By injecting nitrogen and removing CO_2_, the carbonate salt regenerated into the amine form for reuse (Figure 2b). Though the recovery rate for BM was as high as 99.96 ± 1.2%, the extraction rates for BS and concentrated BM were only 7.57 ± 0.59% and 77.27 ± 4.51%, respectively. Aside from the input sensitivity, the amine may interact with dairy components and cause toxic consequences [93], and the chemical facilities required may be incompatible in a dairy factory setting.

MPLs can be dissolved in ethanol and alkanes [21,67,68], and may not dissolve in acetone, ethyl acetate, and 2-pentanone [21,67,68]. Lipid BMP (100 g) dissolved in ethanolic hexane (1:4 *v*/*v*, 800 mL) under constant agitation at 45 °C for 2 h will produce an extract. The permeate of vacuum filtration (repeated twice) can then be vacuum-dried at 1 kPa (Figure 2c). The residue (≈20 g) is then defatted twice with 120 mL acetone, and the resulting acetone is insoluble (AI, ≈7 g), composed of mainly polar lipids, and in the final step vacuum, is dried again at 1 kPa [21]. However, acetone poses a degree of toxicity, as acetone residue in defatted MPLs may reach 5–10 ppm. Further, acetone can form a mesityl oxide via a condensation reaction, causing an off flavor [94]. Hence, toxic residues in acetone-insoluble fractions need analysis and monitoring.

### 3.2. Supercritical Fluid Extraction

**Figure 3:**
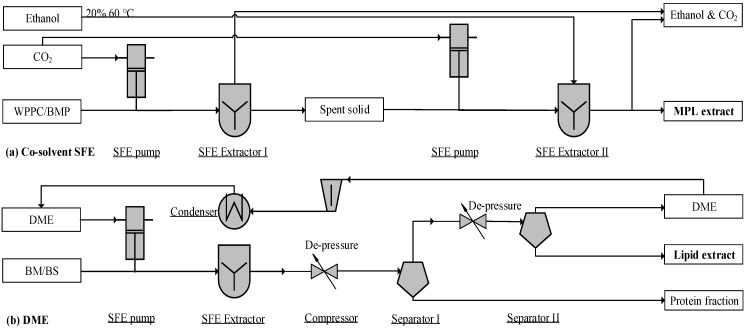
Process flow diagram of a supercritical fluid extraction (SFE) unit: (**a**) adapted from Li [49] and (**b**) Kala et al. [97]; WPPC, whey protein phospholipid concentrate; BS, beta serum; BM, buttermilk; BMP, buttermilk powder; DME, dimethyl ether.

Supercritical CO_2_ with ethanol as a co-solvent can be used to extract MPLs effectively, yielding purities of 26.26% and 16.88% from WPPC and BMP extractions, respectively (Figure 3a). The SFE operation can be conducted at 50–60 °C [95] and 350–550 bar [49]. The SFE co-solvent (CO_2_ and 20% ethanol) allowed for complete extraction of PE, PC, and SM. However, neither PS (i.e., the vital compounds for cognitive function) nor PI were extracted [61,96]. Therefore, the co-solvent method may be an invalid industrial process due to the incompleteness of PS/PI recovery. In addition to co-solvents, dimethyl ether near the critical point (DME, 20%–30% solubility, 333 K, 40 bar) and supercritical CO_2_ are able to dissolve polar and neutral lipids, respectively [59].

Supercritical fluid DME has been used to extract polar lipids, resulting in a yield of 69.1–77.8%. The SFE process shown in Figure 3b can accept both liquid and powder inputs [59,97]. This unit can work with CO_2_ and DME in two-stages, extracting neutral and polar lipids, separately. In addition to a two-step operation, this unit can also operate a single extraction with DME. Near-critical DME dissolves both polar and non-polar lipids in the SFE chamber. Through a two-stage de-pressurization, lipids are separated from the protein fraction, whereas vaporized DME is compressed and condensed for reuse (Figure 3b). This method features properties such as non-toxicity, a compact skid, feeding flexibility, and a high content of MPLs (65.7–75.5 g MPLs per 100 g DM). However, the MPL recovery rate (69.1–77.8%) needs further improvement.

### 3.3. Enrichment of Milk Phospholipids via Filtration

**Figure 4:**
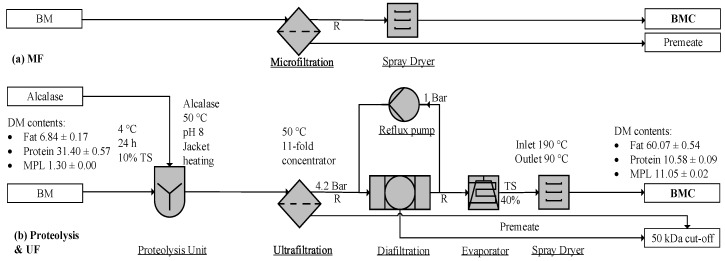
Filtration to enrich milk phospholipids (MPLs): (**a**) microfiltration (MF) [98] and (**b**) ultrafiltration (UF) [96]. BM, buttermilk; BMC, BM concentrate; TS, total solid; R, retentate.

BM or BS is composed of milk fat, casein and whey protein, lactose, and ash. The particle sizes range from 0.4–4 µm for MFGM fragments or phospholipid micelles [98], 0.02–0.3 µm for casein, 0.03–0.06 µm for whey protein, and 0.002 µm for lactose and ash, respectively [99]. The size of MFG is around 0.2–15 µm [3]. As illustrated in Figure 4, the MF unit removes lactose and whey protein, and UF separates the smaller casein proteins from MPLs. Due to the size overlap of casein micelles and MPL particles, their separation is usually incomplete. Casein micelles disintegrate into peptides and amino acids in the proteolysis unit [34,42], and hydrolysates enter into the permeate stream during the subsequent UF operation [42,96]. Alcalase (E.E. 3.4.21.62), a serine-type endoprotease with esterase activity, catalyzes amino esters at pH 7.5 and 35–60 °C [96], while tryptic and peptic hydrolysis may be carried out at 42 °C for 2–16 h, at a pH of 7.7 and 2.0, respectively [42].

Membrane filtration is a typical process for enriching BM (Figure 4a). Proteolytic treatment plus UF, as illustrated in Figure 4b, successfully differentiates MFGM from protein particles and yields product purities of 14 ± 3.4% (DM) [42] and 11.05 ± 0.02% (DM) [96]. The combined process of proteolysis and membrane separation can yield a 100% recovery rate of MPLs from BM, as illustrated in Table 3. Considering membrane units exist in most dairy factories [99,100], the process remains the most effective method for concentrating MPLs, requiring less investment than the other processes [101]. As illustrated in Table 3, this method [96] recovered more MPLs than the other processes.

### 3.4. Available Processes for Extracting Milk Phospholipids

In brief, there are three options for the large-scale manufacturing of MPLs, including solvent extraction [21,68], SFE [59,97], and proteolysis plus membrane concentration [34,42,82,96]. The membrane concentration of MPLs have yielded a 20% (*w*/*w*, DM basis) purity, as achieved by Lecico [58] and Arla [10]. Tatua [56] and Westland and Synlait [44] have extracted MPLs from BS powder (2.28%, *w*/*w*, DM basis), achieving approximately 12.8% (*w*/*w*, DM basis) purity using membrane filtration. The proteolysis and UF unit recovers MPLs completely [34,82,96] and cost-effectively [44]. This process is more efficient than SFE and solvent extraction, whereas SFE and solvent extractions are effective steps for manufacturing high purity MPLs. Therefore, the three processes have features of a high recovery rate, facility availability, and food compatibility, representing current industrial practices (in Table 3).

## 4. Carbon Footprint

### 4.1. Life-Cycle Accessment Method of Carbon Footprint

**Figure 5:**
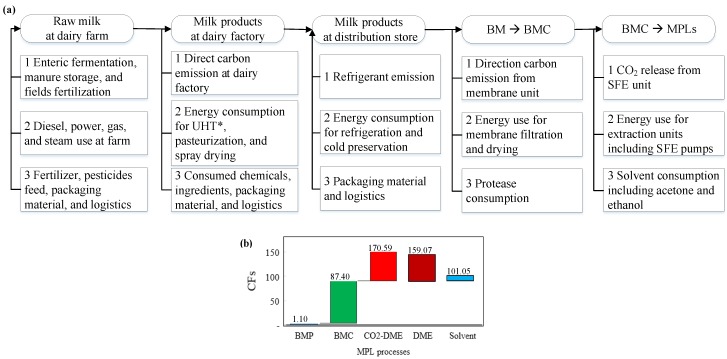
(**a**) Boundary definition of the life-cycle carbon footprints (CFs) of dairy products and exemplary emissions from scope 1 (direction emission), scope 2 (energy carriers), and scope 3 (raw material procured, packaging material, and transportation). (**b**) Cascade of CFs of BMP, BMC, and MPLs using the following processes: “CO_2_-DEM” (supercritical CO_2_ and DME), “DME” (supercritical DME), and “Solvent” (hexane and ethanol extraction and acetone de-fatting, kg equivalent CO_2_/products). Scopes of BM CFs: adapted from References [45,106,107,108,109]. MPLs, milk phospholipids; BM, buttermilk; BMC, BM concentrate; DME, dimethyl ether; SFE, supercritical fluid extraction. * Ultra High Temperature processing.

The ISO 14,040 life-cycle assessment (LCA) is an internationally accepted methodology used to calculate a product’s environmental footprint [103]. Life-cycle carbon footprints (CFs) of dairy products cover the direct emission from the dairy factory (scope 1); the energy carrier footprint for factory operations (natural gas, steam, power, nitrogen, and compressed air in scope 2); and the raw material, packaging, and logistics in scope 3. In addition, the life-cycle CF comprises the emissions from the dairy farm (upstream) and distribution center (downstream) [104]. The boundaries are set as shown in Figure 5a. The CFs of MPL products were reported as equivalent CO_2_ emission for one kg of MPLs, according to the ISO 14,067 reporting standards [105].

The CF of BM (baseline CF, 1.10 kg CO_2_/kg BM powder) was cited directly from data derived from the Unified Livestock Industry and Crop Emissions Estimation System (ULICEES) model in Canada [45]. The data abides by the Intergovernmental Panel on Climate Change (IPCC) methodology [106]; it covers emissions like methane [45], nitrous oxide [107], and carbon dioxide using the F4E2 model [108]; and uses an allocation matrix to partition six inventory flows (i.e., fuel, power, raw milk transportation, alkaline/acid, water, and waste water) into 11 major dairy products [109].

In this study, BM was assumed as the starting material for producing MPLs. Therefore, the CF for producing BM was set as the baseline. The CF of MPLs in Figure 5b and Table 4 is a sum of the CF of BM (as the baseline) and the CF for extracting MPLs from BM at dairy factories. The starting amount of BM was assumed to be 100 kg (1.3%, *w*/*w*, DM basis). Since MPLs are considered as the target products, CF of protein in the MPL fractions was not included in the estimations.

The CF of BM concentrate (BMC) via membrane separation (MS) was calculated using the equation: CF_BMC_ = CF_BM_ + CF_MS_, where CF_BMC_, CF_BM_, and CF_MS_ were the CF of BMC, BM, and MS, respectively. The CF of MPL products using SFE or solvent extraction was calculated using the equation CF_MPLs_ = CF_BMC_ + CF_SFE_ or CF_MPLs_ = CF_BMC_ + CF_Sol_ where CF_MPLs_, CF_BMC_, CF_SFE_, and CF_Sol_ were the CF for the MPL product, BMC, SFE, and solvent extraction process, respectively, as illustrated in Figure 5b. The CF for BMP (1.5% purity) was 1.10 kg CO_2_/kg MPL, and the CF of BMC (11.0% purity) was 87.40 kg CO_2_/kg MPL, as calculated in Table 5. Starting from BMC, the CFs of MPL products were 170.59, 159.07, and 101.05 kg CO_2_/kg MPL for processes of CO_2_-DME supercritical fluid extraction, DME SFE, and organic solvent extraction, respectively.

### 4.2. Carbon Footprint Estimation

In Table 4, four MPL enrichment processes were used as references for estimating and comparing the total CFs. The membrane separation process was used to concentrate MPL from the original BM. The resulting product was a BM concentrate (BMC), which may be further processed to yield MPL products by either using an SFE technique or a solvent extraction method. The CF of “utility” consumed for the three individual MPL enrichment methods was obtained by multiplying the utility amount and CF conversion factor, which represents the amount of carbon emission for a unit weight of utility. Normalized CF: CF_Normalized_ = CF/C_MPLs_, where CMPLs was the MPL purity (g MPLs per 100 g product).

The normalized CF of the product uisng membrane separation was as high as 87.4 kg CO_2_/kg BMC since the BMC comprised of only 11.05% MPLs. The CFs for products using SFE and solvent extraction were much higher than their baseline (CF_BMC_) because of the intensive process during purification. As shown in Table 4, the CFs of fractions using SFE were 170.59 and 159.07 kg CO_2_/kg MPLs for CO_2_/DME co-extraction and DME extraction, respectively. CO_2_/DME co-SFE exhibited a higher environmental impact compared to supercritical DME extraction due to direct emissions from co-SFE. Solvent extraction demonstrated a lower environmental impact and a higher MPL recovery rate than SFE. However, the products obtained using solvent extraction were less food-compatible than SFE unit-extracted products.

MPLs from proteolysis and filtration processes carry 87.40 kg equivalent CO_2_/kg product, much higher than all the milk fat products (Table 5). With less CF than SFE and solvent extraction, membrane separation is the most efficient process in terms of process intensity, energy consumption, and environmental impact. In addition, this process is compatible with most dairy factories. Membrane separation is a necessary step for concentrating BM into BMC. BMC can then be purified using SFE (DME). The relevant processes with a significant MPL CF include membrane filtration, evaporation and spray drying, SFE, and solvent recovery, the improvement of which offer opportunities to reduce the CF of the final products. For example, 0.1-µm polymeric spiral-wound MF membranes have been used to separate casein from milk, exhibiting a higher energy efficiency at 0.024 (MF) and 0.015 (DF) kWh/kg permeate than that of graded permeability membrane (0.143 and 0.077 kWh/kg permeate for MF and DF, respectively [110]. Furthermore, permeate flux, volume concentration ratio, transmembrane pressure, and temperature all had an impact on the energy efficiency of membrane UF, ranging from 0.26–0.33 kWh/kg retentate [116]. Another approach toward reducing the environmental impact is to improve the purity of MPLs during filtration by differentiating the particle size of casein micelles (i.e., hydrolysis) from the fragmented MFGM and subsequent application of membrane filtration.

## 5. Conclusions

This paper identified three dairy streams for milk phospholipid (MPL) manufacturing at an industrial scale: buttermilk, beta serum, and whey protein phospholipid concentrate. The life-cycle CFs of the MPLs were 87.40, 170.59, 159.07, and 101.05 kg CO_2_/kg MPLs for the membrane separation process, CO_2_/DME supercritical fluid extraction, SFE by DME, and organic solvent extraction, respectively. The extracted products comprised 11.1, 76.8, 69.9, and 88.0% MPLs, with recovery rate of 100, 69.1, 67.4, and 100%, respectively. In conclusion, to improve the efficiency of an MPL concentration process, casein in BM needs to be proteolyzed before running UF/DF processes. By doing so, it is possible to achieve full recovery of MPLs from BM; moreover, this method may result in a relatively low CF. SFE using dimethyl ether is the most effective method for the production of high-purity (≈66.8%) MPL products, albeit at the cost of a high CF. This study provided insights into the best available industrial practices for extracting MPLs and estimating their life-cycle CFs.

## Figures and Tables

**Table 1:** Dairy product composition (g/100 g).

Product	Total MPLs	DM MPLs	Fat MPLs	DM Protein	DM Fat	Total Solid	DM Ash	Reference
WPPC	1.60	7.92	29.10	65.00	27.00	20.20	7.92	[48]
WPPC	1.78	1.78	11.63	56.64 ± 0.05	24.23 ± 0.02	97.02	2.57 ± 0.02	[49]
WPPC	2.20	2.20	14.57	64.82 ± 0.12	18.71 ± 0.09	96.40	2.32 ± 0.01	[49]
WPPC	2.20	2.20	14.38	65.00 ± 0.06	18.46 ± 0.01	95.96	2.27 ± 0.03	[49]
BMP	1.30	1.30 ± 0.00	19.01	31.40 ± 0.57	6.84 ± 0.17	-	-	[34]
BM	0.14 ± 0.04	-	-	25.01 ± 0.76	12.22 ± 1.56	-	5.60 ± 0.16	[50]
BM	0.13 ± 0.00	1.43 ± 0.00	25.50	3.46 ± 0.05	0.51 ± 0.02	9.12 ± 0.17	-	[32]
BM	0.16 ± 0.02	1.78 ± 0.17	15.1 ± 0.5	32.44 ± 0.83	11.78 ± 0.53	9.02 ± 0.23	-	[6]
BS	0.97 ± 0.05	8.78 ± 0.41	38.6 ± 2.3	32.41 ± 1.01	22.71 ± 1.04	11.05 ± 0.43	-	[6]
BS	0.93 ± 0.07	8.42 ± 0.63	34.57	3.55 ± 0.11	2.69 ± 0.14	11.05 ± 0.40	-	[8]
BM	0.12 ± 0.01	1.36 ± 0.07	25.36	32.68 ± 0.93	4.87 ± 0.12	8.63 ± 0.26	-	[51]
BS	0.97 ± 0.17	8.8 ± 1.1	40 ± 7	33 ± 3	25 ± 8	11.0 + 0.8	-	[43]
BM	0.11 ± 0.01	1.2 ± 0.1	14 ± 5	33 ± 2	10 ± 5	8.7 ± 0.8	-	[43]
Whey BM	0.16 ± 0.01	2.01 ± 0.16	12.04 ± 0.8	24.89 ± 2.02	16.27 ± 2.06	8.05 ± 0.32	7.01 ± 0.47	[38]
BM454974 ^a^	-	-	-	3.33 ^b^	3.33 ^b^	-	5 ^c^	[41]
BM336087 ^a^	-	-	-	3.21 ^b^	3.31 ^b^	12.09	0.69/4.88 ^c^	[41]
BM171274 ^a^	-	-	-	34.3	5.78	97.03−BMP	7.95	[41]
CM495516 ^a^	-	-	-	3.33	36.67	-	3.33 ^c^	[41]
CM336519 ^a^	-	-	-	2.84	36.08	42.19	2.74 ^c^	[41]

MPLs, milk phospholipids; BS, beta serum; BM, buttermilk; BMP, BM powder; WPPC, whey protein phospholipid concentration; CM, cream; DM, dry matter; ^a^ United States Department of Agriculture (USDA) FoodData Central ID [41]; ^b^ wet basis; ^c^ lactose; Ref., reference.

**Table 2:** Proprietary/patented manufacturing technologies of milk phospholipids (kg/100 kg products).

Applicant	Input	Technology Used	MPL Content	Reference
Fonterra	BSP	SFE CO_2_ defat, hi-pressure DME extract	65.7–75.5	[59]
Meggle	BSP	SFE CO_2_ defat, hi-pressure ethanol extract	≈98.5	[60]
Owen John	BSP	SFE CO_2_ defat, ethanol co-solvent extract	PI/PS lost	[61]
Arla	BSP	MF, ethanol extraction	16–19	[9]
Merchant & Gould	Cream	UF, DF	27.7–38.8	[62]
Marc Boone	BM	UF 5–20 kDa	≈2.84	[63]
Land O’Lakes	BM, BS	UF, defat using SFE CO_2_	>30	[64]
Morinaga	Whey BM	MF 0.2 µm, defat using SFE CO_2_	≈22	[65]
Snow Brand	-	Extract using acidic ethanol, defat	-	[66]
Enzymotec	-	Extract using ethanol & hexane, acetone defat	≈24	[21]
Cargill	-	Extract using alcohol (C_1_–C_3_), acetone defat	-	[67]
Svenska	BMP	Extract using ethanol & n-heptane, acetone defat	≈70 SM	[68]

MPLs, milk phospholipids; BM, buttermilk; BMP, BM powder; BS, beta serum; BSP, BS powder; MF/UF, micro/ultra-filtration; DME, dimethyl ether; PI, phosphatidylinositol; PS, phosphatidylserine; SM; sphingomyelin; SFE, supercritical fluid extraction.

**Table 3:** Process to purify MPLs and achieved purity (g MPLs/100 g dry product).

Reference	Input	Technology Used	Purity	Recovery (%)
[97]	BSP	SFE: CO_2_, 300 bar, 40 °C, DME	12.9 → 75.7 (5.9-fold)	69.1
[97]	BSP	SFE: DME, 40 bar, 50 °C	12.9 → 66.8 (5.2-fold)	62.9
[49]	WPPC	SFE: 350 bar, CO_2_, 20% ethanol, 60 °C	2.2 → 26.3 (11.9-fold)	PS/PI lost
[49]	BMP	SFE: 550 bar, CO_2_, 15% ethanol, 60 °C	2.0 → 16.9 (8.6-fold)	PS/PI lost
[50]	BMC	SFE: CO_2_ defat	2.2 →7.8 (3.5-fold)	100
[50]	BMC	SFE: CO_2_ defat	2.2 → 9.2 (4.2-fold)	100
[98]	BMC	SFE: CO_2_ defat	9.6 → 19.7 (2.1-fold)	100
[38]	BMC	SFE: CO_2_ defat	7.2 → 12.0 (1.7-fold)	100
[93]	BM	Solvent: BM (6:1 *v*/*v*) extraction	-	87.5
[93]	BM	Solvent: BM (12:1 *v*/*v*) extraction	-	99.9
[93]	BS	Solvent: BS (12:1 *v*/*v*) extraction	-	7.6
[42]	Whey BM	Proteolysis, UF/DF, 300 kDa, 40 °C	0.3 → 8.6 (28.7-fold)	95–99
[42]	Whey BM	Proteolysis, UF/DF, 300 kDa, 40 °C	0.4 → 11.4 (27.1-fold)	95–99
[42]	Whey BM	Proteolysis, UF/DF, 300 kDa, 40 °C	0.5 → 14.0 (26.4-fold)	95–99
[96]	BMP	Proteolysis, UF/DF, 50 kDa, 50 °C	1.3 → 11.1 (8.5-fold)	100
[34]	BMP	Proteolysis, UF/DF, 50 kDa, 50 °C	0.8 → 6.2 (7.8-fold)	100
[102]	BM	MF, 0.2 µm	1.5	67
[98]	BM	MF, 0.8 µm	9.6	-
[32]	BM	MF/DF, 0.5 µm, 50 °C	1.4 → 2.5 (1.8-fold)	88.8
[32]	BM	MF/DF, 0.5 µm, 50 °C	1.4 → 4.1 (2.9-fold)	89.7
[50]	BMP	MF/DF, 0.45 µm, 9 °C	1.2 → 2.2 (1.8-fold)	60.87
[50]	BMP	MF/DF, 0.45 µm, 9 °C	1.5 → 2.2 (1.5-fold)	87.34
[50]	BMP	MF/DF, 0.45 µm, 9 °C	0.5 → 0.9 (1.7-fold)	90.12
[50]	BMP	MF/DF, 0.45 µm, 9 °C	0.3 → 0.7 (2.3-fold)	80.24
[35]	CWBM	UF, 0.15 µm cellulose acetate	1.8 → 2.3 (1.3-fold)	41.9
[35]	CWBM	UF, 0.15 µm cellulose acetate, TA	1.8 → 4.7 (2.7-fold)	98.4
[38]	CWBM	UF/DF, 10 kDa	2.0 → 7.2 (3.6-fold)	-
[36]	CWBM	TA, wash at pH 7.25, UF/DF, 55 °C	2.0 → 10.7 (5.4-fold)	>90

BM, buttermilk; BS, beta serum; BMP, BM powder; BSP, BS powder; CWBM, cheese whey BM; WPPC, whey protein phospholipid concentrate; BMC, BM concentrate; DME, dimethyl ether; SFE, supercritical fluid extraction; MF/UF/DF, micro/ultra/dia-filtration; TA, thermal aggregation.

**Table 4:** Normalized carbon footprints of milk phospholipids (kg CO_2_/kg MPLs).

Process	Membrane	SFE (CO_2_/DME)	SFE (DME)	Solvent Extract	Unit
Reference	[96]	[59,97]	[59,97]	[21]	-
Input	BMP	BMC	BMC	BMC	-
Input amount	100.00	100.00	100.00	100.00	kg
Input purity	1.3	5.7	6.8	12.3	g/100 g DM
Product	BMC	MPLs	MPLs	MPLs	-
Product amount	11.76	5.13	6.56	13.98	kg
Product purity	11.05	76.80	66.80	88.00	g/100 g DM
MPL yield	100.00	69.10	67.40	100.00	%
Power	17.48	512.85	655.68	-	kWh
Material used	Alcalase 0.03	CO_2_ 1000.00 DME 200.00	DME 200.00	C_6_/ethanol 552.00 Acetone 189.60	kg kg
Thermal energy	13.10	-	-	-	MJ
Power CF factor	0.1567	0.1567	0.1567	0.1567	kg CO_2_/kWh
Material CF factor	5.00	CO_2_ 0.05 DME 0.16	CO_2_ 0.05 DME 0.16	C_6_/ethanol 0.16 Acetone 0.42	kg CO_2_/kg kg CO_2_/kg
Thermal CF factor	0.06	-	-	-	kg CO_2_/MJ
CF of power	2.74	80.36	102.74	-	kg CO_2_
CF of material	0.16	82.00	32.00	167.95	kg CO_2_
Thermal CF	0.72	-	-	-	kg CO_2_
Utility CF	3.62	162.36	134.74	167.95	kg CO_2_
BM/BMC baseline	110.00	498.17	594.31	1074.99	kg CO_2_
Product CF	9.66	128.80	111.19	88.93	kg CO_2_/kg
Normalized CF	87.40	170.59	159.07	101.05	kg CO_2_/kg MPLs

BM, buttermilk; BMC, BM, concentrate; MPLs, milk phospholipids; C6, hexane; DME, dimethyl ether; SFE, supercritical fluid extraction; UF/DF, ultra/dia-filtration; CF, carbon footprint. Membrane filtration power consumption: 1.486 kWh/kg products [110]; Canada power CF factor: 0.1567 kg CO_2_/kWh [111]; CF of reusable solvents (DME, hexane and ethanol): 0.16 kg CO_2_/kg solvent; reused acetone CF: 0.42 kg CO_2_/kg solvent [112]; DME CF 1.01 kg/kg; 84% reuse [113]; baseline of BM: 1.1 kg CO_2_/kg BMP [45]; SFE CO_2_ reuse 95% [114]; SFE CO_2_/DME power cost estimation 100 kWh/kg extract [115].

**Table 5:** Comparison of the carbon footprint of milk phospholipids in commercial dairy products (kg CO_2_/kg product).

Dairy Products	CF	Scope 1	Scope 2	Scope 3	Country	Reference
Raw milk	1.10	-	-	-	Canada	[45]
Bulk liquid	1.00	0.870	0.065	0.065	Canada	[45]
Yogurt	1.50	1.083	0.252	0.165	Canada	[45]
Whole milk	1.12	0.843	0.173	0.104	China	[117]
Powder milk	10.10	-	-	-	Canada	[45]
Butter	7.30	-	-	-	Canada	[45]
BM	1.10	-	-	-	Canada	[45]
Cheese	12.40	-	-	-	Italy	[104]
Cheese	5.30	-	-	-	Canada	[45]
Cheese	8.80	-	-	-	Sweden	[118]
BM → BMC: UF/DF	87.40	-	-	-	-	[96]
BM → BMC → MPLs: SFE CO_2_/DME	170.59	-	-	-	-	[97]
BM → BMC → MPLs: SFE DME	159.07	-	-	-	-	[97]
BM → BMC → MPLs: Solvent extract	101.05	-	-	-	-	[45]

MPLs, milk phospholipids; BM, buttermilk; BMC, BM concentrate; DME, dimethyl ether; SFE, supercritical fluid extraction; UF/DF, ultra/dia-filtration; CFs, carbon footprints.
